# Road testing new CAR design strategies in multiple myeloma

**DOI:** 10.3389/fimmu.2022.957157

**Published:** 2022-08-09

**Authors:** Priyanka S. Rana, Elena V. Murphy, Jeries Kort, James J. Driscoll

**Affiliations:** ^1^ Division of Hematology & Oncology, Department of Medicine, Case Western Reserve University, Cleveland, OH, United States; ^2^ Department of Biochemistry, Case Western Reserve University, Cleveland, OH, United States; ^3^ Case Comprehensive Cancer Center, School of Medicine, Case Western Reserve University, Cleveland, OH, United States

**Keywords:** CAR T-cell therapy, multiple myeloma, hypoxia, armored CAR, self-driving CAR, logic-gates

## Abstract

A deeper understanding of basic immunology principles and advances in bioengineering have accelerated the mass production of genetically-reprogrammed T-cells as living drugs to treat human diseases. Autologous and allogeneic cytotoxic T-cells have been weaponized to brandish MHC-independent chimeric antigen receptors (CAR) that specifically engage antigenic regions on tumor cells. Two distinct CAR-based therapeutics designed to target BCMA are now FDA-approved based upon robust, sustained responses in heavily-pretreated multiple myeloma (MM) patients enrolled on the KarMMa and CARTITUDE-1 studies. While promising, CAR T-cells present unique challenges such as antigen escape and T-cell exhaustion. Here, we review novel strategies to design CARs that overcome current limitations. Co-stimulatory signaling regions were added to second-generation CARs to promote IL-2 synthesis, activate T-cells and preclude apoptosis. Third-generation CARs are composed of multiple co-stimulatory signaling units, e.g., CD28, OX40, 4-1BB, to reduce exhaustion. Typically, CAR T-cells incorporate a potent constitutive promoter that maximizes long-term CAR expression but extended CAR activation may also promote T-cell exhaustion. Hypoxia-inducible elements can be incorporated to conditionally drive CAR expression and selectively target MM cells within bone marrow. CAR T-cell survival and activity is further realized by blocking intrinsic regulators of T-cell inactivation. T-Cells Redirected for Universal Cytokine Killing (TRUCKs) bind a specific tumor antigen and produce cytokines to recruit endogenous immune cells. Suicide genes have been engineered into CAR T-cells given the potential for long-term on-target, off-tumor effects. Universal allo-CAR T-cells represent an off-the-shelf source, while logic-gated CAR T-cells are designed to recognize tumor-specific features coupled with Boolean-generated binary gates that then dictate cell-fate decisions. Future generations of CARs should further revitalize immune responses, enhance tumor specificity and reimagine strategies to treat myeloma and other cancers.

## CAR T-cells as a strategy to treat multiple myeloma

Multiple myeloma (MM) is hematologic malignacy that lacks curative therapy (1, 2). In 2022, an estimated 34,470 new cases of MM will be diagnosed in the US (19,100 men, 15,370 women) and MM will account for ~12,640 deaths (7,090 men, 5,550 women) (3). Preclinical studies revealed a more precise understanding of the pathobiology of MM that has translated into therapeutic strategies that significantly improved patient quality-of-life and overall survival (OS) (4–6). Since the turn of the century, the myeloma field has witnessed two paradigm-shifting approaches that rapidly yielded clinical benefit and abruptly altered treatment patterns (7). Since plasma cells (PCs) are professional antibody-producing factories, myeloma cells are exquisitely sensitive to proteasome inhibitors (PIs) that disrupt protein homeostasis (8–12). Clinical success of the first U.S. Food and Drug Administration (FDA)-approved PI bortezomib launched a meteoric rise in interest of MM by basic scientists, physicians and the pharmaceutical industry (5–7, 13–15). Recently, immunotherapy in the form of antibodies, antibody-drug conjugates and engineered T-cells has been incorporated into first-line and relapse regimens, to improve OS for newly diagnosed and relapsed and/or refractory MM (RRMM) (14–16). Despite these advances, constitutive genetic complexity combined with an immunosuppressive microenvironment, remain obstacles (17–19). Standard of care therapy for MM patients includes chemotherapy, autologous stem cell transplantation (ASCT), the PIs bortezomib, carfilzomib, and ixazomib, immunomodulatory drugs (IMiDs; thalidomide, lenalidomide, and pomalidomide) and the monoclonal antibodies daratumumab and elotuzumab. Despite the recent FDA approval of >15 therapies, many of those diagnosed with MM develop drug-resistant disease and relapse.

**Figure 1:**
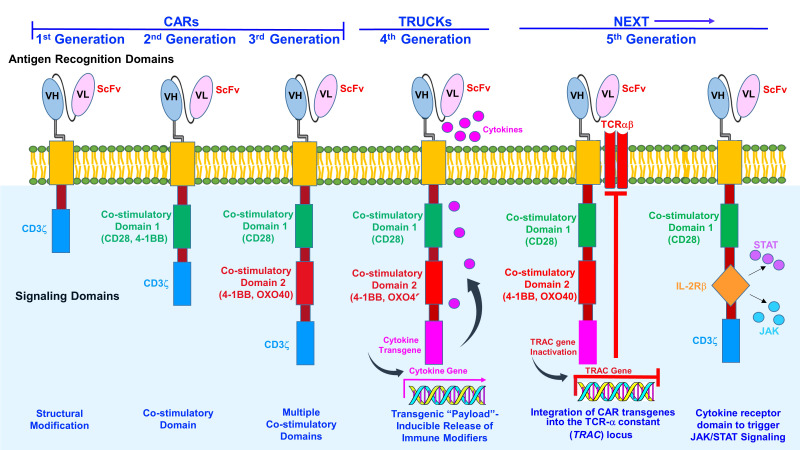
Generations of CAR T-cells. The first-generation CAR T-cells consisted of an intracellular CD3 ζ- chain or FcϵRIγ domain. However, first-generation CAR T-cells did not generate sufficient IL-2 and exogenous IL-2 supplementation was required. In the second- generation, additional signaling domains comprised of T-cell cytokine and costimulatory receptors CAR T-cells were included in the design. Co-stimulatory domains promote IL-2 synthesis to enhance T-cell activation and reduce apoptosis. Third-generation CAR T-cells contain an antigen recognition domain, hinge, membrane-spanning region and a cytoplasmic domain. Third-generation CAR T-cells consist of two co-stimulatory signaling units, e.g., CD28 (B7), CD137 (4-1BB), CD134, (OX40), DAP10, as well as a CD3ζ or FcϵRIγ domain. Third-generation CARs promote cytokine secretion to increase T-cell proliferation and survival. Fourth-generation CARs T-cells (TRUCKs) store transgenic cytokine and release it when induced to attract innate immune cells. Some constructs also incorporate a suicide gene, e.g., *Caspase-9*, to rapidly withdraw CAR T-cells once anti-tumor effects are reached. Two examples of newly emerging fifth (next)-generation CAR T cells are shown. Next-generation (fifth-generation) CAR T-cells integrate CAR transgenes into the TCR-α constant (*TRAC*) locus. CAR can be directed to the *TRAC* locus, resulting in uniform CAR expression, reduced tonic signaling, decreased exhaustion and increased antitumor efficacy and gives the added benefit of producing a potential universal product. To induce JAK-STAT pathway activation in CAR-T cells in an antigen-dependent manner, the full-length or truncated cytoplasmic domain of a membrane receptor, e.g., IL-2 receptor-β can be incorporated between the cytoplasmic domains of CD28 and CD3z. The cytokine receptor domain triggers JAK/STAT signaling to promote proliferative capacity and functional activity.

Chimeric antigen receptor (CAR) T-cell therapies represent a transformative means to revamp immunologic responses and improve the outcomes for difficult to treat malignancies such as RRMM (20–23). CARs are fusion proteins that consist of an antigen-recognizing extracellular single chain variable fragment (scFv) merged with a membrane-spanning region and a cytoplasmic co-stimulatory domain, e.g., the CD3ζ portion of the T-cell receptor (TCR) (23–27, **Figure 1**). CD3ζ promotes T-cell activation, while other co-stimulatory molecules, e.g., CD-28, 41BB, and OX-40, augment T-cell responses. The 4-1BB domain is associated with a memory phenotype to enhance T-cell persistence while CD28 is linked with effector T-cells.

## Mechanisms of resistance to CAR T-cell therapy

Despite impressive responses that have observed in different cancer types, numerous escape mechanisms to evade CAR-T cells have been identified, including antigen escape (through mutation, loss or downregulation), proteolytic cleavage of the target antigen, intratumoral heterogeneity, T-cell exhaustion and cancer immunoediting (28–31). Of the known escape mechanisms that have been identified, the best defined etiology of disease relapse has been due to target antigen loss. Multiple strategies have been proposed to override the limitations of conventional and current approaches that employ CAR T-cells in cancer treatment. Recently reported clinical data indicated that 7–33% of responders in CAR-19 T-cell trials for B-cell acute lymphoblastic lymphoma (B-ALL) relapsed because of the loss of cell-surface CD19 (32). Following CAR-19 T-cell therapy, Sotillo et al. identified acquired mutations as well as alternatively spliced *CD19* alleles in malignant B cells of relapsed patients. This resulted in a lack of CD19 expression or expression of CD19 variants that did not contain the epitope recognized by the CAR T-cells. Fischer et al. suggested that CD19 isoforms lacking the CAR T-cell binding epitope (CD19-negative relapse) are present in some patients prior to therapy, predisposing these individuals to treatment failures (33).

In pre-clinical studies, targeting HER2 in a *glioblastoma multiforme* (GBM) cell line resulted in the emergence of *HER2*-null tumor cells that maintained expression of non-targeted tumor-associated antigens. Combinational targeting of these tumor-associated antigens could counter mechanisms of antigen escape. Hegde et al. reported single-cell co-expression patterns of HER2, IL-13Rα2, and EphA2 in primary GBM samples using multicolor flow cytometry and immunofluorescence (34). The authors applied a binomial routine to the permutations of antigen expression and mathematical modeling demonstrated that co-targeting HER2 and IL-13Rα2 could maximally expand the therapeutic reach of the T-cell product. Schneider et al. also reported results with another CD19–CD20 tandem CAR (35). Constructs were generated where CD19 or CD20 was expressed as the distal receptor on the CAR protein (designated CAR1920 or CAR2019) and compared to single antigen CARs. Both CAR1920 and CAR2019 tandem constructs were superior to a CD19 single-CAR in a murine xenograft model. Lastly, Vie et al. engineered T-cells to express CD16 (FcγRIII) CARs capable of mediating antibody-dependent cellular cytotoxicity (ADCC) (36). Taken together, it is reasonable to speculate that multi-targeting CAR T-cell strategies may overcome the current limitations and improve efficacy.

Relevant to the treatment of MM, the B-cell maturation antigen (BCMA) is detectable on the extracellular membrane of clonal and polyclonal PCs, as well as healthy, memory B cells (37–40). BCMA represents a favorable target for CARs because of exclusivity to cells of the B-cell lineage, prevalence on PCs and promising preclinical results. Interestingly, the degree of expression varies because of γ-secretase-mediated cleavage and shedding of soluble BCMA (sBCMA) into the circulation (41, 42). Shedding of BCMA also indicates that MM can persist without expression of this target. In addition, the biallelic loss of *BCMA* locus represents a mechanism of resistance to therapies targeting this molecule (43, 44).

Since CAR T-cells have been designed to specifically recognize and eliminate BCMA-marked cells, these agents have now been evaluated in clinical trials and yielded high response rates. However, current limitations to therapy include antigen downregulation and escape, T-cell dysfunction, an immunosuppressive tumor microenvironment (TME), unwanted toxicities, and resistance to therapy. CAR T-cell success is exemplified by FDA-approval of the anti-CD19 CAR T-cell tisagenelcleucel for treatment of acute lymphoblastic leukemia (ALL) and axicabtagene ciloleucel for diffuse large B-cell lymphoma (DLBCL). In early 2021, liso-cel received FDA-approval for DLBCL, based upon significant efficacy and low toxicity (45, 46).

## Clinical studies that led to FDA-approval of CAR T-cell therapies for myeloma

The US FDA recently approved the use of idecabtagene vicleucel (ide-cel) in patients with MM who received >4 prior lines of therapy. The ide-cel CAR is comprised of a murine extracellular scFv specific for recognizing BCMA, attached to a human CD8-α hinge and a transmembrane domain fused to the T-cell cytoplasmic signaling domains of CD137 4-1BB and CD3-ζ chain, in tandem. Ide-cel recognizes and binds to BCMA on the surface of MM cells leading to CAR T-cell proliferation, cytokine secretion, and subsequent cytolytic killing of BCMA-expressing cells. In March 2021, idecabtagene vicleucel (ide-cel) was the initial BCMA–directed CAR T-cell immunotherapy approved to treat patients with RRMM based upon results of the KarMMa study (47). The KarMMA study determined the safety and efficacy of ide-cel in heavily pre-treated MM patients *per* International Myeloma Working Group (IMWG) criteria and that did not respond to the last regimen received. Patients who had received >4 previous therapies that included an IMiD, a PI and a CD38-targeting antibody demonstrated a PFS of ~9 months. The overall response rate (ORR) was 73%, median progression-free survival (PFS) was 8.8 months and most subgroups, e.g., high-risk, the elderly, had an ORR of >50%. CAR T-cells were detected in 59% of patients at 6 months and 36% at 12 months following receiving therapy. Adverse events (AEs) of any-grade included cytokine release syndrome (CRS), neutropenia, thrombocytopenia, and neurotoxicity. Importantly, the KarMMa study reported outcomes for 128 of the 140 patients enrolled on the study. Two of the 12 patients who underwent leukapheresis but did not receive CAR T-cell infusion died following lymphodepletion chemotherapy.

Ciltacabtagene autoleucel (cilta-cel) features two BCMA-targeting single domain antibodies and has also been recently FDA-approved for RRMM patients after having received >4 lines of therapy that included a PI, an IMiD, and a monoclonal antibody that targeted CD38 (48). BCMA-positive patients treated with cilta-cel exhibited an ORR of 98%, a stringent complete response (sCR) rate of 80.4% and a very good partial response (VGPR) of 14.4%. The median time to first response was 1 month, median time to best response and CR or greater was 2.6 months and median duration of response was 21.8 months. Over 90% of patients were minimal residual disease (MRD) negative and patients displayed an 18-month progression-free survival (PFS) rate of 66.0%. Patients that achieved a sCR had an 18-month PFS rate of 75.9%. These trials have transformed the treatment armamentarium of RRMM, with unprecedented ORRs in this difficult-to-treat patient population. However, a significant proportion of patients ultimately relapse despite achieving deep remission. MM appears to have emerged as a model system to develop and study novel CAR T-cell design strategies to overcome drug resistant and/or refractory diseases. Several innovative approaches including alternative and dual-antigen–specific CAR T-cell constructs, genetically-engineered off-the-shelf CAR T-cells, and strategies to counteract an immunosuppressive microenvironment may reshape the CAR T-cell field. These strategies are being actively investigated to enhance the durability of responses and extend patient survival.

## Correlation of MM patient baseline characteristics with CAR T-cell response

Baseline cytogenetic abnormalities are associated with unique clinical and immunological characteristics of MM at diagnosis and may influence response to CAR T-cell therapy (49).

Whether these factors are significantly associated with the prognosis of patients undergoing CAR T-cell therapy remains to be fully understood. In a high-risk subgroup analysis from the pivotal KarMMa study, high incidences of response were consistently observed in most subgroups examined, including older patients, and those with more aggressive disease features, including high-risk cytogenetic abnormalities, a high tumor burden, and extramedullary disease (47–51). However, other groups showed poorer PFS for patient receiving anti-BCMA directed CAR-T cell therapy with extramedullary disease, light chain MM and high-risk cytogenetics (TP53 mutation, deletion of 17p13 or gains/amplification of 1q21 (52). A meta-analysis of anti-BCMA CAR T-cell studies associated the presence of high-risk cytogenetics with lower ORRs, whereas extramedullary disease was not associated with reduced response (53). Other cytogenetic mutations are being studied as potential targets for CAR T-cells. The t(14;16)(q32;q23) and t(14;20)(q32;q11) translocations are observed in ~4% and 2% of NDMM patients in which *MAF* and *MAFB* are overexpressed, respectively (54). Ectopic overexpression of large MAFs results in dysregulated expression of downstream genes, including integrin β7 (*ITGB7*). In MM cells, overexpression of *ITGB7* enhances cell adhesion, migration, and invasion, which are related with cell-adhesion-mediated drug resistance (55). MMG49, CAR-T cells targeting the activated ITGB7 protein are a potential therapeutic option for patients with t(14;16) or t(14;20) (56, 57).

Despite the significant improvement in survival outcomes of MM over the past two decades, myeloma remains a nearly universally incurable disease. Patients with MM with triple-class (PI, IMiD, and anti-CD38 monoclonal antibody) refractory status have limited effective treatment options (49). Hence, the development of new therapeutic options for these patients is critical. MM has emerged as a model system to evaluate novel CAR designs and CAR T-cell therapies represent an innovative, personalized and groundbreaking approach to improve myeloma treatment.

## Alternative antigenic proteins for CAR T-cells to target in multiple myeloma

The expression of BCMA is variable and there is a significant risk of relapse due to antigen escape. Hence, there is a need to identify additional surface-associated targets on MM cells. Attractive molecules to target need to be stably expressed with low intratumoral and interpatient heterogeneity, lack expression on other essential tissues and demonstrate negligible antigen solubility (58–60). CARs designed to target CD19, CD38, CD138, SLAMF7 and GPRC5D antigens are in development.

### CD19

CD19 is detected on a subset of myeloma cells (61, 62). It has been postulated that CD19+“stem cells” that propagate myeloma and that these cells can be best targeted following high-dose chemotherapy debulking of the non-CD19+ population.

### CD38

CD38 is expressed at high levels on PCs and preclinical results supports the anti-myeloma effect of CAR T-cells that target CD38 (63). CD38 is similarly detected on healthy erythrocytes, NK cells, and other cell types, increasing the likelihood of “on-target, off-tumor” effects (64).

### CD138

CD138 is found on PCs but also on many normal tissues increasing the risk of off-tumor effects. CAR T-cells that target CD138 were shown to not be toxic to epithelial cells and patients treated with these CAR T-cells did not develop adverse toxicities (65).

### SLAMF7

SLAMF7 (signaling lymphocyte activation molecule F7, CS1) is detected on PCs, healthy B cells, T-cells, NK cells, monocytes, and DCs. CAR T-cells that target SLAMF7 demonstrate promising activity in preclinical studies (66). Since SLAMF7 is also found on many other normal (healthy) cell types, SLAM7-directed CAR T-cells may exhibit fratricide of SLAMF7^high+^ B, T, and NK cells, but spare SLAM7^−/low^ cells.

### GPRC5D

*GPRC5D* expression is >500 times greater on PCs and MM cells than other cell types (67). GPRC5D may represent a novel antigen than can be used alone or in multi-CAR strategies.

### SEMA4A

SEMA4A is a new target for MM and is expressed at a greater level on MM cells than BCMA and SLAMF7. SEM4A is rapidly internalized, displays a low level of shedding and appears essential for MM survival, making it unlikely to be genetically eliminated (68).

## Design strategies to Improve CAR T-cell efficacy in cancer treatment

CAR T-cell designs that incorporate multiple target antigens can be combined with established CAR T-cells, e.g., BCMA, to increase tumor lysis, prevent antigen escape in order to improve overall efficacy and sustain clinical responses. Potential combinations include anti-BCMA CAR T-cells with CD19, CD38, SLAMF7, and GPRC5D (69–76). Dual CAR T-cells can be either administered simultaneously by infusion of two pools of T-cells that express separate CARs or by infusion of a single T-cell pool in which two T-cell populations express separate CARs. Conceivably, dual targeting by distinct CAR T-cells is advantageous since both CAR T-cell products can be controlled separately. However, generating two separate batches of CAR T-cells could be an expensive and time-consuming process. Dual target specificity can be accomplished using bispecific tandem CAR constructs or ligand-based CARs to target distinct antigens, e.g., APRIL-CAR (73).

### Targeting multiple tumor cell surface proteins

Targeting more than one antigen receptor can be accomplished by generating two or more cell populations expressing different CARs and infusing them together or sequentially; a bicistronic vector that encodes two different CARs on the same cell; simultaneously engineered T-cells with two different CAR constructs (co-transduction), which generates three CAR T-cell subsets consisting of dual and single CAR-expressing cells; or two CARs on the same chimeric protein using a single vector, i.e., bi-specific or tandem CARs. CD19-positive relapse is usually associated with limited persistence, low potency of CARs, low response to CARs in patients, and transient B-cell aplasia. The CAR co-stimulatory domain influences the persistence of CAR T-cells. The anti-CD19 scFv used in clinical research is mostly murine-derived, which might result in CAR T-cell exhaustion in patients due to its high antigenicity. Recently reported studies support the sequential administration of CAR T-cell therapies (77–79). Sequential CD19-22-20 CAR-T cell therapies demonstrated 41.7% overall CR rate in 17 pediatric patients with r/r B-cell lymphoma, including 13 Burkitt lymphoma (77). For the non-remission patients who received the prior CD19 CAR T-cells, CD20 and CD22 CAR T-cells were subsequently infused on day 30, and three of six patients further achieved CR. With extended duration of CAR-T cell expansion which was contributed by separated expansion of each kind of CAR T-cells, 70.6% overall CR rate was further achieved in six months after traditional sequential CAR-T cell therapies. Sequential infusions of CD20 and CD22 CAR T-cells significantly improved the prognosis of the B-NHL patients, while some advanced patients still progressed to death during these CAR-T cell treatments (78). It was hypothesized that sequential CAR-T cell infusions may induce co-expansion of different CAR T-cells when residual prior CAR T-cells still remain detectable in PB, which leads to the prolonged duration of peak expansion of CAR T-cells with enhanced antitumor effects (79). Sequential infusions of different CAR T-cells enhanced anti-tumor effects, which is consistent with the synergistic effects of multi-agent immunotherapies on eradicating disease and prolonging remission in the patients with relapsed hematologic malignancies. Taken together, the results demonstrate that short-interval sequential infusion of different CAR T-cells can augment CAR-T cell expansion and enhance the anti-tumor effects *in vitro*, in animal models, and in two patients with advance B-cell lymphomas. The broad applicability of sequential infusion of CAR T-cells remains to be determined.

### Dual CAR T-cell therapy

Another approach separates primary activation from co-stimulatory signaling events using separately expressed CARs directed towards two distinct antigens. Splitting the T-cell activation signals enables tumor specificity since dual CAR-transduced T-cells can only be activated fully if both CARs simultaneously target tumor cells but not if they recognize only one of the antigens on healthy tissues. Another form of dual CAR T-cell therapy uses inhibitory CARs (iCARs) to improve efficacy and safety of myeloma-directed CAR T-cells where a second-generation CAR is combined with an iCAR (80, 81).

### Armored CAR T-cells

Armored CAR T-cells are devised to overcome immunosuppressive activities elicited by the TME. The type of ‘armor’ is predicated on the cytokine milieu of the TME and the roles of innate and adaptive immune cell types that are present. Armored CAR T-cells have been constructed to permit constitutive and/or inducible secretion of active cytokines, express specific ligands, and secrete antibody-like peptides to improve T-cell proliferation and survival (82, 83). These types of CAR T-cells are engineered to be resistant to immune suppression and could more likely be modified in the future to no longer express immune checkpoints.

### T-cells redirected for universal cytokine killing

T-cells Redirected for Universal Cytokine Killing (TRUCKs) are fourth-generation CAR T-cells that, upon binding a specific tumor antigen, specifically produce cytokines designed to attract certain immune cells while suppressing other cell types (84–86). TRUCK CAR T-cells represent a specific type of armored CAR T-cell which secretes cytokines to interfere with the immunosuppressive cytokine profile within the solid tumor. TRUCKs are specifically armored to co-express a CAR as well as a cytokine that improves T-cell growth, survival and expansion while simultaneously providing resistance to immunosuppression. TRUCK-driven cytokines also promote the removal of antigen-negative tumor cells by bystander T-cells. Cytokines employed in TRUCKs thus far include IL-8 (87), IL-9 (88), IL-11 (89, 90), IL-12 (91, 92), IL-15 (92), IL-21 (93) and IL-18 (94). Transgenic production of IL-15 (95) and IL-21 further enhances the effects by stimulating innate immunity. IL-23 secreting CAR T-cells increase therapeutic effects with less adverse toxicities relative to IL-15 and IL-18-expressing CAR T-cells. Pro-inflammatory signaling from bone marrow (BM) promotes tumor growth to suggest that TRUCKs may require regulated cytokine release to decrease inflammation and preserve responses.

Transforming growth factor-beta (TGF-β) is a pleiotropic factor produced within the BM niche that promotes tumor initiation, progression and the emergence of drug resistance (96, 97). TGF-β suppresses T-cell immunosurveillance, drives T-cell differentiation towards regulatory T-cells (Tregs) and TGF-β levels correlate with poor prognosis in cancer patients. TGF-β elicits an inhibitory effect on cytotoxic T-cell proliferation, activation, and effector functions to subvert T-cell immunity through Foxp3-dependent and independent mechanisms by favoring Treg differentiation. TGF-β also confers resistance to CAR T-cells and promotes T-cell exhaustion and senescence. CAR T-cells can be created to secrete cytokines that negate immunosuppression within the BM niche. CD28-ζ CAR T-cells secrete IL-2 that offsets the immune suppressive effects of TGF-β. Inhibitory cytokines can also be eliminated by increasing expression of IFN-γ and IL-12 which leads to more effective tumor clearance (98).

It has been proposed that BCMA CAR T-cells can be “armored” to repel TGF-β-mediated immunosuppression. B2ARM CAR T-cells were developed to co-express a BCMA-specific CAR together with a TGF-β dominant-negative receptor type II, in CD4+ and CD8+ T-cells (99). B2ARM CAR T-cells effectively targeted myeloma (MM.1S) cells, whereas myeloma killing activity of B2CAR T-cells without armor was blocked by the addition of TGF-β. Moreover, following exposure to TGF-β, B2ARM CAR T-cells demonstrated an increase in Ki67. The serine protease granzyme is commonly found in granules within T-cells that are secreted along with pore-forming perforin to mediate apoptosis in target cells. Granzyme B levels were also greater in B2ARM CAR T-cells, as was CD107a. B2ARM CAR T-cells also blocked TGF-β-driven changes in the levels of CD25, PD-1, LAG3 (exhaustion markers), and CD45RA+ CD45RO-CD62L- (differentiation markers). B2ARM CAR T-cells improved the survival of NSG mice that harbored RPMI-8226 tumors and overexpressed TGF-β. B2ARM CAR T-cell infused mice displayed greater infiltration of tumors 7 days after treatment, and greater IFN-γ, TNF-α, Ki67, granzyme B, and PD-1 expression relative to tumor-infiltrating non-armored B2CAR T-cells. Mice also received RPMI-8226 cells in which exogenous TGF-β was administered and mice that received B2ARM CAR T-cells rejected tumors faster than mice that received non-armored B2 CARs. Mice that received B2ARM CAR T-cells also demonstrated a higher number of CD3+ and CD3+CAR+ cells as well as central memory and effector memory T-cells. Armored B2ARM CAR T-cells appear to promote enhanced persistence, increased survival, greater differentiation of effector cells and superior anti-myeloma activity. Armored B2ARM CAR T-cells abrogated a number of TGF-β-driven functional activities that suppress anti-myeloma immunity (100–102).

### CAR T-Cells that target tumors under hypoxic conditions

Tumor cells responses to low oxygen (O_2_) concentrations are mediated primarily through hypoxia-inducible factors that modulate transcriptional changes (103–105). In myeloma, higher proliferative rates, greater metabolic needs, and insufficient vasculature to meet tumor demands, yield an O_2_ deficient environment, measured to be <2% compared with healthy tissues where the O_2_% is ~20% (106–108). In cancer patients, a hypoxic TME has been linked with resistance to chemotherapy, radiotherapy and CAR T-cells as well as an overall poor prognosis. Since hypoxia differentiates BM from normoxic tissues, hypoxia-inducible CARs may represent an attractive signaling system to selectively induce the expression of antigen recognizing molecules on the myeloma surface.

Since hypoxia is a characteristic of the myeloma TME, studies were performed to determine the effects of low O_2_ concentrations (hypoxia) on CAR T-cell growth, proliferation and differentiation (109). CD19 and BCMA-specific CAR T-cells were cultured under atmospheric (18% O_2_) and hypoxic (1% O_2_) conditions. CAR T-cells cultured in 1% O_2_ expanded much less than cells cultured at 18% O_2_, were less differentiated and had an increased the ratio of CD4 to CD8. CAR T-cells cultured under atmospheric or hypoxic conditions were then added to antigen-^pos^ or antigen-^neg^ tumor cells and displayed comparable cell-killing activities with upregulation of PD-1. In contrast, the production of cytokines and granzyme B release were lower under hypoxic conditions, even CAR T-cells generated under atmospheric conditions. Since hypoxia modulates tumor growth and immune editing, a dual oxygen-sensing switch was designed to provide stringent hypoxia-dependent regulation of a CAR (110). The microdevice platform mimics the 3-D tumor with an O_2_ gradient that facilitates evaluation of CAR T-cell anti-tumor infiltration and efficacy.

### Self-driving CAR T-cells

CAR T-cells utilize potent promoters to enforce long-term CAR expression, but constitutive CAR activation may promote T-cell exhaustion. To overcome this limitation, self-driving CAR T-cells were generated that regulate their own function with the help of signal transducer promoters. CD19-targeting CAR engages the antigen controlled by the activator protein 1 (AP1)-nuclear factor kappa−B (NF-κB) or signal transducer and activator of *STAT5* promoters (111). Self-driving CAR T-cells exhibit comparatively low T-cell exhaustion *in vitro* and offer a solution to limitations imposed by CAR-T cell persistence.

### Suicide gene inactivation of CAR T-cells

Genetically-engineered overexpression of immune stimulatory cytokines requires built-in safeguards to prevent potential toxicities. The introduction of a conditional suicidal phenotype or a safety switch into allogeneic CAR T-cells can enhance the safety profile and facilitate future clinical development and applications. Since *SLAMF7* is expressed on healthy leukocytes, especially NK cells that control viral infections, inclusion of a suicide gene with an anti-SLAMF7 CAR is prudent (112). In general CAR T-cell depletion, as another OFF/safety-switch for cell-based therapies can be realized by constructing CARs that express a suicide gene, e.g., *inducible delta Caspase 9* (*i-delta-Casp9*) (113–117). Constructs that harbor an *IL-15* and *i-delta-Casp-9*-based suicide gene (*iC9/CAR.19/IL-15*) also offer potential (118). T-cells that expressed *i-delta-C9/CAR.19/IL-15(+)* demonstrated more expansion upon stimulation, reduced cell death rate, lower PD-1 receptor expression and enhanced anti-tumor efficacy. Activated T-cells obtained from either normal (healthy) donors or acute myeloid leukemia (AML) patients were treated with retroviral supernatant that encoded inducible *i-delta-Casp9*, a ΔCD19 marker for selection, and a CAR that recognized CD33. T-cells that express *iC9-CAR.CD33* may be included as part of the conditioning therapy for ASCT in AML, since *Casp9* activation would remove genetically-modified T-cells before the infusion of stem cells to reduce engraftment failure. *Casp9* fused to the FK506 binding protein (FKBP) is a safety switch that allows for conditional dimerization. However, activation of the Casp9 domain of iCasp9 depends on dimer formation of FKBP12 domains, achieved using the drug rimiducid (AP1903) (119).

### Logic-gated CAR T-cells

**Figure 2:**
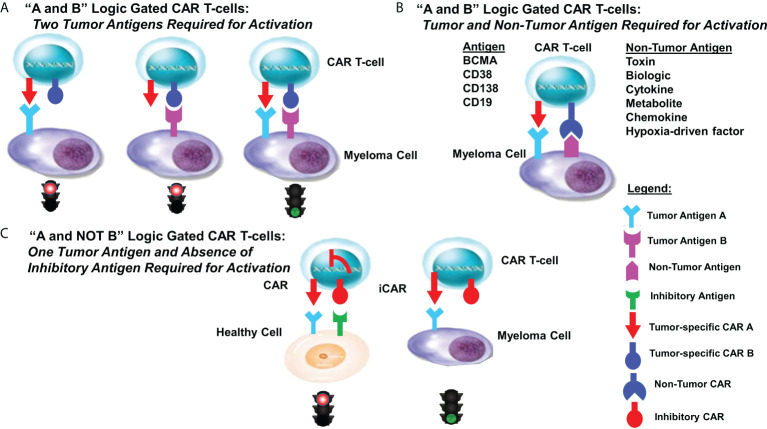
Logic-gated CAR T-cell design strategies. Shown are various designs for logic-gated CAR T-cells. **(A)** “A and B” logic-gated CAR T-cell design “A and B” Logic Gated CAR T-cells: *Two Tumor Antigens Required for Activation.* Tumor cells express two distinct tumor-specific antigens. CAR T-cells express two separate CARs that each are required to recognize and bind a single tumor antigen. Binding of a single antigen alone does not promote CAR T-cell activity. The two distinct CARs are co-expressed with complementary signaling domains in one T-cell that fully activates the T-cell only in the presence of both cognate antigens. **(B)** “A and B” Logic Gated CAR T-cells: *Tumor and Non-Tumor Antigens Required for Activation.* Tumor cells express one tumor-specific antigen as well as a non-tumor antigen. CAR T-cells are designed to express two separate CARs that each are required to recognize and bind a single antigen. Binding of a single antigen alone does not promote CAR T-cell activity. **(C)** “A and NOT B” Logic Gated CAR T-cells: *One Tumor Antigen and the Absence of* an *Inhibitory Antigen Required for Activation.* Inhibitory CARs (iCARs) are not able to adequately activate cytotoxic activity upon recognition and binding cells that express one of the two targeted antigens. iCAR-T cells selectively kill target cells that express only one antigen, whereas healthy (off-target) cells co-expressing another inhibitory ligand recognized by the iCAR are protected, allowing T-cells to distinguish target cells from healthy (non-tumor) cells.

Computationally designed assembly of recombinant proteins allows for the programmable control of protein-protein interactions that function as tunable, molecular sensors to govern critical cellular activities and dictate cell fate decisions (120–122). A strategy for the design of more specific and effective CARs is to merge CAR T-cells that target more than one antigen equipped with “AND”, “OR” and “NOT” Boolean-derived logic-gates, i.e., on/off switches that produce a single binary output. Logic-gated CARs perform a specific functional operation triggered by one or more physical or chemical input signals linked by an output transistor to downstream elements that activate a defined output (**Figure 2**). Simultaneous targeting of a second protein or co-factor triggers a selective response and represents an “AND” binary decision logic-gate. The premise that CARs can respond to more than one feature specific to the cancer cell represents a rational approach to develop more efficacious “on-target, on-tumor” therapies. Given the limits of most cancer drugs, one approach is to continue to identify and target unique features of myeloma cells in order to design more specific CARs (22, 123–125).

## Loss of MRD negativity as a general mechanism of disease relapse

MM patients frequently attain a BM MRD negativity status in response to treatment (126). Time from ASCT revealed patients with MRD conversion during the first three years had inferior OS and PFS compared with patients with sustained MRD negativity. MRD conversion correctly predicted relapse in 70%, demonstrating the utility of serial BM MRD assessment to complement standard laboratory and imaging to make informed salvage therapy decisions. CAR T-cell therapy is also highly effective in the treatment of B-ALL or B-cell lymphoma, providing alternative therapeutic options for patients who failed to conventional treatment. However, up to 60% patients relapse, probably due to persistence of CAR T-cells and escape or downregulation of CD19 antigen, which is a great challenge for disease control. For CD19-negative relapse, CD19 is absent, causing tumors to evade CAR recognition and clearance in spite of CAR T-cell persistence. Jacoby et al. found in murine studies that immune pressure rather than immune selection of CD19 by CAR T-cells led to the reprogramming of the B-ALL lineage, resulting in late relapse (127).

Trogocytosis is a process in which lymphocytes extract surface molecules from antigen-presenting cells (APCs) through immunological synapses (128). Intriguing studies have shown that CAR T-cells extricate and acquire target antigens into T-cells through trogocytosis. A minimum density of the targeted antigen at the cell surface appears necessary for T-cells to elicit cytotoxicity. Trogocytosis may reduce antigen density below the minimum threshold needed to promote T-cell activity. Combinatorial strategies to target more than one antigen may permit next-generation CARs to prevent or overcome tumor escape caused by trogocytosis.

## Conclusions and perspectives

A biologic rationale for the evolutionary development of mechanisms that elicit cell-mediated cytotoxicity within multicellular organisms remained a quandary for decades (129). While multicellular organisms depend on cooperation and communication between different cell types, it was not clear why sensitized lymphocytes produced cytopathogenetic and cytolytic effects on homologous cells *in vitro*, in the absence of classical humoral antibodies (130). It is now understood that cell-mediated cytotoxicity is directed against syngeneic cells that bear infectious agents or have been mutated through oncogenic transformation. Sacrificing a population of defective cells ultimately improves the survival probability of the entire organism. In the 1960’s, a number of studies collectively demonstrated cell-mediated cytotoxicity *in vitro* primarily using combinations of allogeneic cells (131–136). The cumulative results demonstrated that lymphoid cells specifically promoted cytotoxicity with a context similar to that of graft-versus-host disease. Subsequently, in 1976 Morgan *et al.* showed that T lymphocyte growth selectively occurred when fractionated BM cells were incubated in conditioned medium from lymphocytes that had been stimulated with phytohemagglutinin (137). Interleukin-2 was described as a “T-cell growth factor” that induced the proliferation of antigen-stimulated T-cells. IL-2 addition also promoted T-cell expansion and maintained T-cell functional activities. The administration of IL-2 in clinical trials lead to durable, complete tumor regression in patients with solid tumors (138, 139). Subsequently, in 1989 Gross et al. pioneered the first CAR design (a “double-chain” CAR: TCR*a*V_H_+TCRβV_L_) followed in 1993 by Eshhar et al. with the first scFv genetically engineered CAR in T-lymphocytes that were redirected to a target, to combine antibody-specificity with T-cell cytotoxicity (132, 133). The introduction of co-stimulatory signals mimic the natural series of steps that occur when T-cells recognize an antigen (134–136). Collectively, a number of seminal studies over the past seven decades have led to the development of T-cell-centric approaches which have been translated into superior anti-cancer and anti-myeloma therapies (62, 136–147).

The paucity of accurate biomarkers to detect early disease, presentation of disease at later stages and intratumoral heterogeneity collectively reduce the impact of conventional treatments in oncology. Chemotherapy, surgery and radiotherapy provide little benefit for many patients that present with relapsed and/or refractory disease, while in contrast, immunotherapeutics have shown significant promise. A number of immunotherapeutics recently have been FDA-approved or are in development to treat MM when administered alone or in novel combinations (148). Resistance to anti-myeloma therapy, in particular to the PIs, inevitably emerges and is a leading cause of relapsed and/or refractory disease and a number of mechanisms have been proposed to characterize PI resistance which is characterized by slowly proliferating, drug tolerant cells that display cross-resistance to numerous PIs (149).

CAR T-cell therapies may potentially provoke life-threatening complications, require careful patient selection criteria as well as a clinical team experienced in the administration of autologous and allogeneic cell therapies. Moreover, responses using currently available CAR T-cells are frequently not sustained. Genomic heterogeneity, drug-induced selection and molecular evolution of tumor clones increase the difficulty to design universally effective CAR T-cells for myeloma. Disease relapse of antigen-expressing tumor cells appears to be tightly related to CAR T-cell durability and exhaustion. T cells from the BM of MM patients were more severely impaired than peripheral T cells and displayed features of exhaustion and senescence. T cells from MM patients are able to recognize and eliminate myeloma, although this is subverted in the majority of patients who eventually succumb to progressive disease. T cell exhaustion and a suppressive bone marrow microenvironment have been implicated in disease progression, and once these are established, immunotherapy appears largely ineffective. In summary, although MM remains largely incurable despite the development of second-generation novel agents and the introduction of monoclonal antibodies, next-generation CAR design strategies should yield more potent T-cell phenotypes that reinvigorate anti-myeloma immunity (150–155).

## Author contributions

JD, PR, EM and JK developed the concept, wrote, edited, made substantial contributions and approved the final version of the manuscript. All authors contributed to the article and approved the submitted version.

## Funding

Research was supported by NIH R01 (5R01AI139141 to JD), University Hospitals Cleveland Medical Center/Seidman Cancer Center, and the Case Comprehensive Cancer Center.

## Conflict of interest

The authors state that the manuscript was prepared without any commercial or financial relationships and without any conflict of interest.

## Publisher’s note

All claims expressed in this article are solely those of the authors and do not necessarily represent those of their affiliated organizations, or those of the publisher, the editors and the reviewers. Any product that may be evaluated in this article, or claim that may be made by its manufacturer, is not guaranteed or endorsed by the publisher.

## Glossary

**Table:**

ADCC	Antibody-dependent Cellular Cytotoxicity
AEs	Any-Grade Adverse Events
ALL	Acute Lymphoblastic Leukemia
AML	Acute Myeloid Leukemia
AP1	Activator Protein 1
APCs	Antigen-presenting Cells
ASCT	Autologous Stem Cell Transplantation
B-ALL	B-cell acute lymphoblastic lymphoma
BCMA	B-Cell Maturation Antigen
BM	Bone Marrow
CAR	Chimeric Antigen Receptors
cilta-cel	Ciltacabtagene Autoleucel
CI	Confidence Interval
CRS	Cytokine Release Syndrome
DLBCL	Diffuse Large B-Cell Lymphoma
FDA	Food and Drug Administration
FKBP	FK506 Binding Protein
GBM	*glioblastoma multiforme*
GPRC5D	G-Protein Coupled Receptor Family C Group 5 Member D
iCasp9	inducible Caspase 9
ide-cel	Idecabtagene Vicleucel
IMiD	Immunomodulatory Drugs
iCARs	Inhibitory CARs
IFN-γ	Interferon Gamma
IL	Interleukin
IMWG	International Myeloma Working Group
LAG3	Lymphocyte Activating 3
MM	Multiple Myeloma
MRD	Measurable Residual Disease
NF-&kappa;B	Nuclear Factor Kappa-B
ORR	Overall Response Rate
OS	Overall Survival
PC	Plasma Cell
PD-1	Programmed Cell Death Protein 1
PFS	Progression-Free Survival
PI	Proteasome Inhibitors
RRMM	Relapsed and/or Refractory Multiple Myeloma
SEMA4A	Semaphorin-4A
SLAMF7	Signaling Lymphocyte Activation Molecule F7
scFv	Single-Chain Variable Fragment
sBCMA	Soluble B-Cell Maturation Antigen
sCR	Stringent Complete Response
STAT5	Signal Transducer and Activator of Transcription 5
TCR	T-cell Receptor
TRUCK	T-cells Redirected for Universal Cytokine Killing
TGF-&beta;	Transforming Growth Factor-beta
TME	Tumor Microenvironment
TNF-&alpha;	Tumor Necrosis Factor-Alpha
VGPR	Very Good Partial Response
